# An Economical Tandem Multiplex Real-Time PCR Technique for the Detection of a Comprehensive Range of Respiratory Pathogens

**DOI:** 10.3390/v1010042

**Published:** 2009-06-08

**Authors:** Glenys R. Chidlow, Gerry B. Harnett, Geoffrey R. Shellam, David W. Smith

**Affiliations:** 1 Department of Microbiology, Pathwest Laboratory Medicine WA, QEII Medical Centre, Nedlands, WA 6009 Australia; 2 School of Biomedical, Biomolecular and Chemical Sciences M502, University of Western Australia, Nedlands, WA 6009 Australia, E-Mails: gerry.harnett@health.wa.gov.au (G-B.H); gshellam@cyllene.uwa.edu.au (G-R.S.); david.smith@health.wa.gov.au (D-W.S)

**Keywords:** respiratory pathogens, tandem multiplex real-time PCR, mixed infections

## Abstract

This study used real-time PCR assays to screen small sample volumes for a comprehensive range of 35 respiratory pathogens. Initial thermocycling was limited to 20 cycles to avoid competition for reagents, followed by a secondary real-time multiplex PCR. Supplementary semi-nested human metapneumovirus and picornavirus PCR assays were required to complete the acute respiratory pathogen profile. Potential pathogens were detected in 85 (70%) of pernasal aspirates collected from 121 children with acute respiratory symptoms. Multiple pathogens were detected in 29 (24%) of those samples. The tandem multiplex real-time PCR was an efficient method for the rapid detection of multiple pathogens.

## 1. Introduction

In recent years, improved nucleic acid amplification and detection technology has facilitated the identification of pathogens that had previously proved difficult or impossible to detect using traditional culture or immunofluorescent techniques. The increased use of molecular methods has also resulted in the discovery of novel respiratory pathogens including the human coronaviruses NL63 [1], HKU1[2] and the SARS coronavirus [3], human metapneumovirus [4], human bocavirus [5] and two human polyomaviruses, KI [6] and WU [7]. Despite these advances, there remain a significant proportion of respiratory disease episodes for which a pathogenic agent can not be identified [8, 9].

The aim of this study was to modify real-time PCR assays to facilitate the rapid screening of respiratory samples for a comprehensive range of viral and bacterial pathogens. Standard PCR assays require extraction of RNA and DNA with simultaneous removal of inhibitors of the PCR process from the sample. Multiple extracts from each sample have been required to set up a comprehensive range of PCR assays for respiratory pathogens, and the sample volume available may be insufficient for all of the required tests. Also, previous attempts at multiplex PCR have been limited by competition between different amplification reactions and by the limited number of fluorescent probes able to be monitored in the same tube. A multiplex tandem PCR assay which utilized a two step PCR system consisting of a limited multiplex PCR followed by a single target PCR has been reported [10]. Our study further modified that procedure to include real-time PCR with specific probes in the second step of the assay. It comprised two first round enrichment PCR assays (A and B) containing 27 and 7 primer pairs respectively, and the second step real-time PCR assays containing 1 – 3 primer pairs and specific Taqman probes. The assay detects several adenovirus genotypes, *Bordetella* species, human bocavirus, *Chlamydophila pneumoniae* and *psittaci*, coronaviruses OC43, 229E, HKU1 and NL63, *Haemophilus influenzae*, influenza viruses A, B and C, *Legionella longbeachae* and *pneumophila*, *Moraxella catarrhalis*, *Mycoplasma pneumoniae*, parainfluenzaviruses 1 – 4, *Pneumocystis jirovecii*, KI and WU polyomaviruses, respiratory syncytial virus types A and B, *Streptococcus pyogenes* and *pneumoniae*. Supplementary semi-nested human metapneumovirus and picornavirus PCR assays are required to complete the acute respiratory pathogen profile.

This technique facilitates the investigation of a comprehensive range of respiratory pathogens from a single nucleic acid extract and a single reverse transcription reaction. In addition, the ability to amplify multiple targets efficiently aids in the detection of mixed infections.

## 2. Results and Discussion

### 2.1. Relative Sensitivity of the tandem multiplex real-time assay

The sensitivity of the tandem multiplex real-time PCR assay was greater than or equal to that of singleplex real-time or traditional nested PCR assays for the majority of the agents investigated (Table 1). Exceptions included the detection of adenovirus and influenza virus C in the tandem multiplex real-time assay, which were 100-fold and 10-fold less sensitive respectively than the established nested PCR assay. While the adenovirus real-time assay performed similarly in the multiplex and singleplex assays, both had lower sensitivity than the traditional nested assay, suggesting the need to redesign the primers and probes used in the real-time assays. This differed from the influenza virus C assay, where the lower sensitivity appeared to be related to the multiplex format, and may therefore necessitate further fine-tuning of the assay.

### 2.2. Clinical samples tested in the tandem multiplex real-time assay

Pernasal aspirates from 121 hospitalized children aged between 3 days and 16 years (mean = 20 months old, median = 7 months old) collected from July to September 2006 were tested in the tandem multiplex real-time PCR assay and the results are presented in Table 2. Respiratory pathogens were detected in 85 samples (71%) and multiple pathogens were detected in 29 of those 85 samples (34%). This detection rate excludes samples where *Moraxella catarrhalis* and/or *Streptococcus pneumoniae* were detected, as the significance of the detection of these agents in pernasal aspirates using PCR amplification techniques remains doubtful [21–23]. Further studies are under way to gather more information about asymptomatic nasopharyngeal carriage rates for viral and bacterial pathogens.

Respiratory syncytial virus (RSV) was the most common pathogen and was detected in 41 samples. For 21 of those samples, at least one, and up to three other respiratory agents, including human rhinovirus, human bocavirus, KI and WU polyomaviruses, human coronaviruses, parainfluenzavirus and group A streptococcus, were also detected. Rhinoviruses were the next most commonly detected (37 samples) and again, multiple agents were commonly detected (17 samples) and included RSV, human coronaviruses, human bocavirus, parainfluenzavirus, adenovirus, KI and WU polyomaviruses and group A streptococcus. Other pathogens detected included influenza virus type B, human metapneumovirus and *Pneumocystis jirovecii*.

The tandem multiplex real-time assay failed to detect pathogens that had previously been detected on initial routine testing in six samples. The agents were rhinovirus (2), parainfluenza type 2, parainfluenza type 3, human metapneumovirus and influenza B. Repeat testing of samples indicated that for the two parainfluenzavirus failures there had been a slight reduction in nucleic acid extraction sensitivity due to the deterioration of carrier RNA used in the extraction process, and once that was overcome the samples performed as expected in the multiplex tandem assay. There was insufficient sample from the remaining four patients to complete similar further investigations. Other samples with discrepant results were due to the absence of specific primers and probes to those agents in this tandem multiplex real-time assay. They included non-group B adenovirus (9), CMV (3) and non-serogroup b *Haemophilus influenzae* (2).

The tandem multiplex real-time assay detected respiratory pathogens in 18 specimens for which no agent had previously been detected. The agents included RSV (4), rhinovirus (7), human bocavirus (3), coronaviruses (3), and parainfluenzavirus. A further 25 specimens had multiple agents detected in the tandem multiplex real-time assay compared to the original test result. These additional detections were mostly due to the comprehensive range of respiratory pathogens detected by the tandem multiplex real-time assay compared to the selective range of tests originally performed as requested by the clinician. Positive results were confirmed by repeat testing, and no-template controls were included after every fifth sample during the extraction and PCR process to detect contamination events.

The samples in this study were collected between June and September 2006, which represents mid-late winter and early spring months, which have typically been the peak respiratory virus detection months in the temperate zones of Western Australia. Despite the comprehensive range of agents detected by the assay described in this study, 35 (29%) samples still failed to yield a positive result. Some of these may be due to suboptimal specimen types for some viruses; poor collection, transport or storage conditions; samples collected too late in the time course of infection; or non-infectious causes of respiratory symptoms. However, it may also indicate possible infection with as yet unknown pathogens.

The significance of the recently described KI and WU polyomaviruses as pathogens remains to be established since, although there has been an association with the respiratory tract [24], a recent report found no association between polyomavirus infection and respiratory disease [25]. In our study KI and WU polyoma viruses were each detected in 4 different samples (3%), but always in combination with another virus, including RSV, adenovirus, rhinovirus, bocavirus, coronavirus and parainfluenzavirus.

The widespread and increasing use of molecular detection techniques has led to reports of multiple pathogenic agents detected in single samples [26–28]. This study supports those findings with two agents detected in 19 (16%) samples, and three or more agents detected in 10 (8%) samples (Table 2). No data was available to determine whether this may have altered the severity of illness. Some studies have reported more severe clinical presentation in the presence of mixed infection [9,29,30] while others have reported no significant greater disease severity in dual respiratory infection [31].

It has been reported that the detection of multiple agents in a multiplex PCR assay was severely compromised when the ratio of target materials in the sample exceeded 100:1 [32]. We also found similar inhibition in a traditional multiplex which was less evident in the tandem multiplex real-time assay (data not shown). In practice, the tandem multiplex real-time assay detected multiple pathogens in 29/121 (24%) samples.

### 2.3. Economies delivered by the tandem multiplex real-time PCR format

Traditionally, testing for a comprehensive range of respiratory pathogens has necessitated multiple PCR assays, immunofluorescence or multiple cell culture assays to be performed on each sample. Considerable specimen volume is required to produce sufficient nucleic acid extracts for multiple PCR assays and, since many respiratory viruses have an RNA genome, multiple reverse transcription reactions are required. Multiple extraction and reverse transcription reactions significantly increase the cost of the assays. There have been recent reports of limited multiplex PCR assays [26,33–36] and real-time multiplex PCR assays for the detection of respiratory viruses and bacteria [37–39]. Immunofluorescent antigen detection tests may be used but require a substantial volume of sample, as well as needing an adequate cellular content. Cell culture techniques require considerable sample volume, a range of different cell lines, and the means for detecting and identifying those agents that are successfully cultivated. Many of the recently described novel viruses including human metapneumovirus, coronaviruses, human bocavirus and some rhinoviruses are difficult or impossible to cultivate in cell cultures [26–28,40,41].

A multiplex tandem PCR was recently described for gene expression profiling [10]. The first enrichment reverse transcription PCR contained up to 72 primer pairs, and the individual second PCR assays utilized SYBR Green detection of products. We have modified that technique to include specific Taqman probes in the second PCR assay to permit specific real-time detection of the PCR product. The two enrichment PCR mixes contain 27 and 7 primer pairs, and the real-time PCR mixes contain 1–3 primer pairs with their corresponding fluorophore-labelled probes (Table 3). The enrichment PCR was limited to 20 amplification cycles to reduce amplification competition between targets, and the dilution of that enrichment PCR product prior to addition to the real-time PCR mix, limited non-specific product formation and carry-over primer competition in the secondary PCR assay [10].

The use of nucleic acid extraction and liquid handling robots streamlined this tandem multiplex real-time assay. The nucleic acid extracted from the specimens and controls was eluted into tubes in a 96-well format. The enrichment PCR and dilutions were all performed in 96-well plates, and robots used to transfer diluted enrichment PCR products to the 100-tube discs preloaded with real-time PCR reaction mixes (Genedisc-100, Corbett Life Science, Australia). Since the initial cycling step was limited to 20 cycles the transfer of those products had a reduced the risk of contamination compared with a traditional nested assay.

The addition of standardized amounts of equine herpesvirus and MS2 RNA coliphage to the digest buffer supplied in the nucleic acid extraction kit, served as a control for the DNA and RNA extraction process, the removal of inhibitors to the cDNA production and PCR amplification processes, and the successful completion of the cDNA procedure. CT values in the specific real-time PCR assays for these internal controls were monitored to avoid false-negative reports.

This tandem multiplex real-time PCR assay, in combination with the semi-nested picornavirus and human metapneumovirus PCR assays, tests for 35 respiratory agents from a sample volume of 180μL compared to 720 μL required for the individual assays. A single sample extraction and three reverse transcription reactions are required for the tandem multiplex real-time assay, compared to four extractions and 17 reverse transcription reactions for the individual assays. In our laboratory the consumable costs are approximately AUD 67 for the individual assays compared to AUD 17 for the tandem multiplex real-time assay. Further work is planned to incorporate the picornavirus and human metapneumovirus assays into the tandem multiplex real-time PCR assay, but so far we have been unable to design or use published real-time PCR primers and probes that detect all types of these viruses with the same sensitivity as our nested assays.

The multiplex tandem PCR assay described has the potential to test for a much larger number of infectious agents, or to look for multiple targets in single agents. In this study, enrichment PCR-B contained only seven primer pairs, so it will be possible to add extra primers to detect a broader range of known or newly described agents. For example, it is planned to include primers directed against adenoviruses other than group B, and a broader range of *Haemophilus influenzae* serogroups. Similarly, continued experience with the assay and further assessment of the role of novel agents in respiratory disease, may lead to the removal of some primer pairs from the PCR mixes.

This test also allows the flexibility to determine which of the organisms included in the enrichment PCR are to be included in the second round, usually with the aim of restricting the initial testing to the most common pathogens. If this is negative, tests for other organisms included in the enrichment PCR can then be performed without the need for more sample or further cDNA synthesis.

## 3. Materials and Methods

### 3.1. Nucleic acid extraction

Bacterial and viral RNA and DNA was extracted from respiratory specimens including nose swabs, throat swabs, pernasal aspirates and sputum samples, using a modified liquid sample protocol with the X-tractor Gene instrument (Corbett Life Science, Australia). Standardized doses of equine herpesvirus type 4 (EHV4) and MS2 RNA coliphage (MS2) were added to the lysis buffer supplied with the kit to monitor the efficiency of sample extraction, removal of reverse transcription and PCR inhibitors, and cDNA production [42, 43]. The CT values expected for the EHV4 and MS2 assays were between 15 and 20, and samples with CT values greater than 27 were retested.

### 3.2. Primer and Probe Design

Taqman primers and probes listed in Table 3, were designed in-house using Primer Express software (Applied Biosystems, USA), with the exception of those for the influenza virus A matrix gene [44]. The letter in the final column of the table indicates which of the enrichment PCR mixes contained the primers, and the number indicates which of the real-time PCR mixes contained the primers and probes. At present, not all of the real-time PCR mixes contain uniquely specific fluorophore-labelled probes, so further confirmation of some positive results is required. For example, in the parainfluenza mixes the types 1 and 2 probes both have FAM labels and the type 4A and 4B probes both have VIC labels, so individual real-time PCRs may be performed from the original enrichment PCR material if further typing is required. The mixes were prepared in large volumes, stored in single use aliquots at −80°C, and batches were quality tested prior to use.

### 3.3. PCR

Enrichment PCR assays A & B, were set up in 96-well plates (Axygen Scientific, USA) using a liquid handling robot (CAS1200 Corbett Life Science, Australia) to add PCR reaction mix and the sample or control nucleic acid extracts. The first enrichment PCR used the Superscript III Platinum One-step RT-PCR system (Invitrogen, USA). The 50μL reaction mix A contained 54 primers each at 0.2–0.3μM, 10 units RNaseOUT (Invitrogen, USA), 10mM DTT (Sigma, USA), 0.75μL Superscript III RT/Platinum Taq Mix, 1.25unit iStar Taq DNA polymerase (Intron Biotechnology, USA), 1.5% DMSO (Sigma, USA), and 8 μL RNA/DNA specimen extract. The cycling conditions were 30 min at 50 °C, 5 min at 95 °C, followed by 20 cycles of 94 ° C for 30 sec, 50 °C for 30 sec, and 68 °C for 45 sec, on an ABI 2700 thermal cycler (Applied Biosystems, USA). The 50 μL reaction mix B contained 14 primers each at 0.2 μM, 4 mM MgCl_2_ (Applied Biosystems, USA), 200 μM dNTP (Fisher Biotec, Australia), 0.01% bovine serum albumin (Sigma, USA), 1.5 units Amplitaq Gold DNA polymerase (Applied Biosystems, USA) and 8μL RNA/DNA specimen extract. Cycling conditions were 95°C for 10 min, followed by 20 cycles of 95°C for 30 sec, 50°C for 30 sec and 72°C for 45 sec.

The initial enrichment PCR products were diluted 1:10 in PCR grade water, and a 1.0 μL sample was added to a 20 μL reaction mix in 100 tube rings (Gendisc-100, Corbett Life Science, Australia) containing 2–6 oligonucleotide primers at 0.2 μM, 0.2 μM Taqman probes, 6 mM MgCl_2_, 0.4 mM dNTP, and 1.0 unit of i-StarTaq DNA polymerase. The dilution and product transfer procedures were performed by a liquid handling robot (CAS1200 Corbett Life Science, Australia). The reaction was cycled on a Rotorgene 6000 real time thermocycler (Corbett Life Science, Australia). The cycling conditions were 94 °C for 2 min, followed by 35 cycles of 94 °C for 12 sec, 55 °C for 90 sec and 72 °C for 20 sec. Probe emission signals were acquired during the 20 sec extension step of the cycling programme. The relatively long 90 second annealing step was found to improve the sensitivity of the multiple primer and probe real-time PCR assay (data not shown).

The singleplex real-time PCR assays used in the sensitivity comparison with the tandem multiplex real-time assays were performed as for the enrichment PCR assays described above, except that the only a single primer pair and the appropriate fluorophore-labelled oligonucleotide probe (0.2 μM) were included in the PCR reaction mix. The number of cycles was increased to 50, the denaturation, annealing and extension steps were reduced to 10, 15 and 15 secs, respectively, and performed on the Corbett RG6000 thermocycler (Corbett Life Science, Australia).

The nested PCR assays used in the sensitivity comparison assays are mostly “in-house-modified” assays and have been used in our diagnostic laboratory for many years. Since they are slowly being superseded by real-time amplification assays, the primer sequences have not been listed in this article but are available on application to the authors. Briefly, the nested assays used 8μL of nucleic acid sample in a 20 μL reaction volume in the Superscript III Platinum One-step RT-PCR (Invitrogen, USA) or the Amplitaq Gold DNA polymerase system (Applied Biosystems, USA). Appropriate enzyme activation was followed by 45 cycles of 95 °C for 30 sec, 50 °C for 30sec and 72 °C for 45 sec. Similar conditions were used for the second PCR assay with 0.5 μL of the initial PCR product as the target DNA. Amplicons were detected by ethidium bromide agarose gel electrophoresis.

The picornavirus and human metapneumoviruses were detected using traditional semi-nested PCR assays as we have not yet been able to design real-time PCR assays to reliably detect all strains of those viruses. The picornavirus PCR utilized published primer sets targeting the 5’ untranslated region of the virus genome [45, 46] and enterovirus or rhinovirus identification was confirmed by DNA sequencing. The in-house human metapneumovirus assay targets the matrix protein gene of the virus.

Well characterized DNA and RNA control materials were tested in traditional nested PCR assays, individual real-time assays and in the multiplex tandem real-time PCR assay.

A collection of 121 respiratory samples from hospitalized children was tested in the multiplex tandem real-time assay. The samples were received at the PathWest Laboratory Medicine WA facility for the routine investigation of respiratory infection and had previously been tested for a narrow range of respiratory pathogens. Samples had been stored at −20°C.

No-template controls were included in the extraction process between every five samples and treated as samples for the completion of the assays. Since all PCR mixes were quality checked for all agents prior to use, and the internal controls cover the extraction, inhibitor removal and cDNA production processes, specific positive control materials were only included in the real-time Taqman PCR assays. Those control materials were well-characterized enrichment PCR products stored in aliquots at −20 °C.

## 4. Conclusions

A tandem multiplex real-time assay is presented which detects a comprehensive range of respiratory pathogens from a specimen sample of 180μL. The initial enrichment PCR eliminates the requirement for individual reverse transcription steps for each of the RNA viruses, resulting in considerable cost and specimen volume benefits. The secondary multiplex real-time PCR utilizes probes labeled with different fluorophores to facilitate a rapid result.

The technique may be used in the routine diagnostic setting or in an outbreak or pandemic situation, to facilitate testing for a range of common, exotic and emerging pathogens. Extra primers and probes for novel agents may be easily accommodated in this technique.

## Figures and Tables

**Table 1.** Relative sensitivity of the multiplex tandem real-time PCR compared to single real-time and nested PCR in-house assays.

**Target DNA/RNA**	**Highest 10 fold dilution detected**		

	**Multiplex Tandem real time PCR**	**Singleplex real time PCR**	**Nested PCR [11–20]**

Adenovirus (Group B)	10^−4^	10^−4^	10^−6^

Bocavirus	10^−7^	10^−7^	NA^b^

*Bordetella pertussis*	10^−5^	10^−5^	10^−5^
*Bordetella holmesii*	10^−8^	10^−8^	10^−8^
*Bordetella parapertussis*	10^−8^	10^−7^	NA
*Bordetella bronchoseptica*	10^−8^	10^−8^	NA

*Chlamydophila pneumoniae*	10^−4^	10^−5^	10^−4^

*Chlamydophila psittaci*	10^−6^	10^−6^	10^−5^

Coronavirus 229E	10^−8^	10^−7^	10^−7^

Coronavirus HKU1	10^−2^	10^−2^	NA

Coronavirus NL63	10^−2^	10^−3^	NA

Coronavirus OC43	10^−8^	10^−9^	10^−8^

*Haemophilus influenzae*	10^−5^	10^−5^	NA

Influenza virus A H1^a^	10^−6^ (Matrix 10^−6^)	10^−6^ (Matrix 10^−6^)	10^−6^

Influenza virus A H3^a^	10^−8^ (Matrix 10^−6^)	10^−7^ (Matrix 10^−6^)	10^−6^

Influenza virus B	10^−6^	10^−7^	10^−6^

Influenza virus C	10^−4^	10^−5^	10^−5^

*Legionella longbeachae*	10^−4^	10^−4^	10^−3^

*Legionella pneumophila*	10^−5^	10^−4^	10^−3^

*Moraxella catarrhalis*	10^−5^	10^−5^	NA

*Mycoplasma pneumoniae*	10^−5^	10^−5^	10^−5^

Parainfluenzavirus 1	10^−8^	10^−7^	10^−8^
Parainfluenzavirus 2	10^−6^	10^−6^	10^−6^
Parainfluenzavirus 3	10^−6^	10^−5^	10^−5^
Parainfluenzavirus 4A	10^−6^	10^−6^	NA
Parainfluenzavirus 4B	10^−3^	10^−3^	NA

*Pneumocystis jirovecii*	10^−3^	10^−2^	10^−2^

Polyomavirus KI	10^−1^	10^−1^	NA
Polyomavirus WU	10^−5^	10^−3^	NA

RSV A	10^−7^	10^−7^	10^−7^
RSV B	10^−4^	10^−4^	10^−4^

*Streptococcus pyogenes*	10^−6^	10^−6^	NA

*Streptococcus pneumoniae*	10^−3^	10^−3^	NA

Equine herpesvirus 4^c^	10^−5^	10^−5^	NA

MS-2 RNA coliphage^c^	10^−8^	10^−7^	NA

a Separate assays for the HA and matrix gene sequences. Matrix assay results in brackets;

b NA = Test not available in this laboratory;

c Viruses added to samples to monitor successful nucleic acid extraction, cDNA production and PCR inhibitor removal.

**Table 2.** Agents detected in PNA samples from 121 hospitalized children.

**Number of samples**	**Original reported result†**	**Tandem multiplex real-time PCR plus supplementary picornavirus and metapneumovirus semi-nested PCR result**

12	RSV	RSV A (4), RSV B (8)
1	RSV, PIV-1	RSV B, PIV-1
12	Human rhinovirus (HRV)	HRV
1	HRV, PIV-1	HRV, PIV-1
4	Influenza virus B	Influenza virus B
1	PIV-1	PIV-1
2	PIV-3	PIV-3
1	Human metapneumovirus	Human metapneumovirus (hMPV)
31	Neg	Neg
4	Neg	RSV A (2), RSV B (2)
7	Neg	HRV
3	Neg	HBoV
1	Neg	HRV, KI polyomavirus
1	Neg	HCoVHKU1
1	Neg	HCoVNL63, *Pneum jirovecii*, WU polyomavirus
1	Neg	HCoVNL63
1	Neg	PIV-3
4	RSV	RSV A, HRV
1	RSV	RSV B, HRV
2	RSV	RSV A, HBoV, GAS
1	RSV	RSV A, HBoV
1	RSV	RSV B, HBoV
2	RSV	RSV A, HRV, KI polyomavirus
2	RSV	RSV B, HCoVHKU1
1	HRV	HRV, HCoVNL63
1	HRV	HRV, HBoV
1	AdV	HRV, AdV
1	Influenza virus B	Influenza virus B, HCoVHKU1
1	RSV, AdV	RSV A, HRV, HBoV, GAS
1	RSV, AdV	RSV B, HRV, HBoV, GAS
1	RSV, AdV	RSV B, HRV, HBoV, HCoVNL63
1	RSV, AdV	RSV A, GAS
2	RSV, AdV	RSV B
1	RSV, AdV	RSV A, HRV, KI polyomavirus
1	RSV, AdV	RSV B, HBoV
1	RSV, *Haemophilus influenzae*	RSV A
1	AdV	WU polyomavirus
1	CMV	HRV, WU polyomavirus
1	CMV	HRV
1	CMV	hMPV
1	*Haemophilus influenzae*	Neg
1	RSV, PIV-2	RSV B
1	RSV, PIV-3	RSV A, HBoV, HCoVHKU1,WU polyomavirus
1	Influenza virus B	Neg
2	HRV	Neg
1	hMPV	Neg

† Original reported result for tests requested by clinician, performed as nested PCR assays except CMV (real-time PCR) and *Haemophilus influenzae* (bacterial culture).

**Table 3.** Primers and probes included in the multiplex tandem real time PCR assay.

Target (Abbreviation) Gene target	Primer or Probe name	Sequence 5′-3′	Size (bp)	PCR mix^a^

Adenovirus (AdV) (Group B) Hexon gene	ADB-F ADB-R ADBProbe1 ADBProbe2	GACAGGATGCTTCGGAGTACCT TTTCTAAACTTKTTYCCCAYAYTGAA 6FAM-CACCAGACCCGGACT-MGBNFQ 6FAM-CTGCACCAGACCCGG-MGBNFQ	91	A-2

Bocavirus (HBoV) VP1	BOCA-F BOCA-R BocaProbe	TGGGCCATTTAATCCACTTGA AATTGAGCAGCGCGATCAG VIC-ATTTACAGGTTCACCGTT-MGBNFQ	63	A-2

Bordetella species Insertion element	IS481-F IS481-R IS481 Probe	CGGATGAACACCCATAAGCA AACTTGATGGGCGATCAATTG 6FAM-TCCTACGTCGACTCGAA-MGBNFQ	81	A-5
*B parapertussis & B bronchoseptica* Pertussis toxin	BPA-F BPA-R BPA Probe	ATCCCGCTACTGTAATCCAA GGTACCATCGTGCGACTTT 6FAM-CACGGCGCAAAC-MGBNFQ	64	A-5

*Chlamydophila pneumoniae* MOMP	CPD-F CPD-R CPD Probe	GAAGGGTTCCATGCAGTTAAGTTT TGATGCTGATAACATCCGCATT VIC-CTCAGCCAAARCTACCTACAGMGBNFQ	75	A-3

*Chlamydophila psittaci* MOMP	CPSITT-F CPSITT-R CPsittProbe	CTCCTTACAAGCCTTGCCTGTAG CCCACATAGTGCCATCGATTAA CY5.5C+CCAGC+TGAA+C+CAAG+TT-BHQ3 (LNA)	68	A-2

Coronavirus 229E (HCoV229E) Nucleocapsid	229E-F 229E-R 229E Probe	CTGCCAAGAGTCTTGCTCGTT TCTTTTCCACCGTGGCTTTT VIC-AGAACAAAAGCATGAAATG-MGBFQ	80	A-6

Coronavirus HKU1 (HCoVHKU1) ORF 1a/b	HKCOR-F HKCOR-R HKCoProbe	CCCGCAAACATGAATTTTGTT CATTCATTCGCAAGGCGATA 6FAM-AATCTATCACCATGTGAA-MGBNFQ	61	A-7

Coronavirus NL63 (HCoVNL63) Nucleocapsid	NL63-F NL63-R NL63 Probe	AACCTCGTTGGAAGCGTGTT CGAGGACCAAAGCACTGAATAA VIC-ATTTTCCTCTCTGGTAG-MGBNFQ	61	A-7

Coronavirus OC43 (HCoVOC43) Nucleocapsid	OC43-F OC43-R OC43 Probe	GACATGGCTGATCAAATTGCTAGT GCTGAGGTTTAGTGGCATCCTT 6FAM-TCTGGCAAAACTTGG-MGBNFQ	67	A-6

Equine herpesvirus 4 (EHV) Glycoprotein gB	EHV-F EHV-R EHV Probe	GATGACACTAGCGACTTCGA CAGGGCAGAAACCATAGACA 6FAM-TTTCGCGTGCCTCCTCCAG-BHQ1	81	B-17

*Haemophilus influenzae*Serotype b capsulation locus	HINF-F HINF-R HINF Probe	AGAAGTTTTACTGATGATATGGGTACATCT GCTCGAAGAATGAGAAGTTTTGTG 6FAM-TTCGCCATAACTTCATCT-MGBNFQ	79	B-14

Influenza virus A Matrix [44]	FA-MATF FA-MATR FA Probe	CTTCTAACCGAGGTCGAAACGTA GGTGACAGGATTGGTCTTGTCTTTA CALOTCAGGCCCCCTCAAAGCCGAGBHQ1	155	A-4

Influenza virus A H3 haemagglutinin	H3N2-F H3N2-R H3HAProbe	ACGAAGTGGGAAAAGCTCAATAAT GGAGTGATGCATTCAGAATTGC 6FAM-ATGCACCCATTGGC-MGBNFQ	72	A-11

Influenza virus A H1 haemagglutinin	H1N1-F H1N1-R H1HAProbe	AAGCTCATGGCCCAACCA CCATTATGGGAGCATGATGCT 6FAM-ATACTCCGGTCACGGT-MGBNFQ	57	A-12

Influenza virus B matrix	FBMAT-F FBMAT-R FB Probe	TGCCTACCTGCTTTMMYTRACA CCRAACCAACARTGTAATTTTTCTG 6FAM-TGCTTTGCCTTCTCCA-MGBNFQ	75	A-4

Influenza virus C NS1	FLUC-F FLUC-R FLUCProbe	CCTAGAACITGGGAAGAKGCR GCAGAATCGTYCCGTTGAA 6FAM-AGRAGCTCACMAYCTTTT-MGBNFQ	61	A-4

*Legionella longbeachae* mip gene	LLong-F LLong-R LLongProbe	TGGTCACTGCGGCCATTA CATCAGTTGCAGCCATTGCT 6FAM-ACATTGCCAAACCCA-MGBNFQ	58	A-3

*Legionella pneumophila* mip gene	LPN-F LPN-R LPN Probe	CAATGGCTAAAGGCATGCAA TGCTGTTCGGTTAAAGCCAAT 6FAM-AGCGCCACTCATAGCGT-MGBNFQ	61	A-3

*Moraxella catarrhalis* OMP	MOR-F MOR-R MOR Probe	TCGCCAAGGTGCRAAAATTAA GCCGTGCTTTCGTCTTTTTC VIC-ACCAATGTTGTTACCTTRCG-MGBNFQ	63	B-14

MS-2 RNA coliphage Coat protein	MS2-F MS2-R MS2 Probe	GTCGACAATGGCGGAACTG TTCAGCGACCCCGTTAGC CALOACGTGACTGTCGCCCCAAGCAACTT-BHQ1	66/74	A-1

*Mycoplasma pneumoniae* *Cytadhesion P1*	MPN-F MPN-R MPN Probe	AAGTACCACCACGACGCTCAA TCGTCAGGGCGGGTGTAG 6FAM–CGCCAGCAATTTA–MGBNFQ	54	A-10

Parainfluenzavirus (PIV) Nucleoprotein	PF1-F PF1-R PF1 Probe PF2-F PF2-R PF2 Probe PF3-F PF3-R PF3 Probe PF4A-F PF4A-R PF4A Probe PF4B-F PF4B-R PF4B Probe	GCAAAGAGARAATGCRGATCTAG AGCTCCGAGACATGCAGGAT 6FAMTCCATATGTCTGAAGCAATMGBNFQ ATTCCAGATGCTCGATCAACTATG TCYTCAGCTAATGCTTCRAARGC 6FAM-AGCACYTCTCCTCTGG-MGBNFQ CGCGCTCCWTTYATCTGTATC TTGCCTGGTGCGAACTCA VIC-TCAGAGATCCYATACATG-MGBNFQ CACACACACAATGGGCACAA GCGTATTTGGTGAGAGTTTTGAGTT VIC-CAATCCCACACTACAAC-MGBNFQ CGCACACATACAATGAAAGCAA TGGCGGGATTTCTGAGTTG VIC-CACTCCTGTCCCACATC-MGBNFQ	67	A-8
			65	A-8
			59	A-8
			65	A-9
			60	A-9

*Pneumocyctis jirovecii* 5S rRNA	PN-F PN-R PN Probe	CCATACCTCAGAGAATATACCGTATCC TACTGACGACGCCCTTCAGA 6FAM–TRACTTCGCAGATCG–MGBNFQ	69	A-10

Polyomavirus VP1	KI-F KI-R KI Probe WU-F WU-R WU Probe	GGTTCTGGAGCTGCCATAGC AAGAAGTGCAGCTCCCTCTGTT 6FAM-CCAAGGGCAGCTAAGCCTTCACCA-BHQ1 AGTCAACCCACAAGAGTGCAAA CAGCACGTCTACCCCTCCTTT VIC-CCTTCCAAAACAAGTCAG-MGBNFQ	77	B-16
			63	B-16

Respiratory syncytial virus A (RSV A) Nucleoprotein Respiratory syncytial virus B (RSV B) Nucleoprotein	RSVA-F RSVA-R RSVAProbe RSVB-F RSVB-R RSVBProbe	CAACTTCTGTCATCCAGCAAA TGCACATCATAATTAGGAGTATCAAT 6FAM-CACCATCCAACGGAGC-MGBNFQ ATTCAACGTAGTACAGGAGATAATA CCACATAGTTTGTTTAGGTGTTT VIC-TGACACTCCCAATTAT-MGBNFQ	77	A-9
			74	A-5

Group A Streptococcus speB gene	STAspeB-F STAspeB-R STspBProbe	CTAAACCCTTCAGCTCTTGGTACTG TTGATGCCTACAACAGCACTTTG 6FAM-CGGCGCAGGCGGCTTCAAC-BHQ1	77	B-11

*Streptococcus pneumoniae* Pneumolysin gene	Pneum-F Pneum-R Pn Probe	CCACTCTTCTTGCGGTTGATC GCCAAACCAGGCAAATCAAT 6FAM-TGCTCCGATGACTTATA-MGBNFQ	61	B-14

a The letter indicates in which of the two enrichment PCR assays the primers are included, and the number indicates in which of 16 real time PCR mixes the primers and corresponding probes may be found.
